# Evaluation of the agreement of tidal breathing parameters measured simultaneously using pneumotachography and structured light plethysmography

**DOI:** 10.14814/phy2.13124

**Published:** 2017-02-13

**Authors:** Shayan Motamedi‐Fakhr, Richard Iles, Anna Barney, Willem de Boer, Jenny Conlon, Amna Khalid, Rachel C. Wilson

**Affiliations:** ^1^ PneumaCare Ltd.ElyUK; ^2^ Cambridge University Hospitals NHS Foundation TrustCambridgeUK; ^3^ ISVRFaculty of Engineering and EnvironmentUniversity of SouthamptonSouthamptonUK

**Keywords:** Bland–Altman, IE50, pneumotachography, structured light plethysmography, tidal breathing, validation

## Abstract

Structured light plethysmography (SLP) is a noncontact, noninvasive, respiratory measurement technique, which uses a structured pattern of light and two cameras to track displacement of the thoraco–abdominal wall during tidal breathing. The primary objective of this study was to examine agreement between tidal breathing parameters measured simultaneously for 45 sec using pneumotachography and SLP in a group of 20 participants with a range of respiratory patterns (“primary cohort”). To examine repeatability of the agreement, an additional 21 healthy subjects (“repeatability cohort”) were measured twice during resting breathing and once during increased respiratory rate (RR). Breath‐by‐breath and averaged RR, inspiratory time (tI), expiratory time (tE), total breath time (tTot), tI/tE, tI/tTot, and IE50 (inspiratory to expiratory flow measured at 50% of tidal volume) were calculated. Bland–Altman plots were used to assess the agreement. In the primary cohort, breath‐by‐breath agreement for RR was ±1.44 breaths per minute (brpm). tI, tE, and tTot agreed to ±0.22, ±0.29, and ±0.32 sec, respectively, and tI/tE, tI/tTot, and IE50/IE50_SLP_ to ±0.16, ±0.05, and ±0.55, respectively. When averaged, agreement for RR was ±0.19 brpm. tI, tE, and tTot were within ±0.16, ±0.16, and ±0.07 sec, respectively, and tI/tE, tI/tTot, and IE50 were within ±0.09, ±0.03, and ±0.25, respectively. A comparison of resting breathing demonstrated that breath‐by‐breath and averaged agreements for all seven parameters were repeatable (*P* > 0.05). With increased RR, agreement improved for tI, tE, and tTot (*P* ≤ 0.01), did not differ for tI/tE, tI/tTot, and IE50 (*P* > 0.05) and reduced for breath‐by‐breath (*P* < 0.05) but not averaged RR (*P* > 0.05).

## Introduction

The importance of measuring tidal breathing became apparent over 60 years ago when clinical differences between healthy subjects and those with respiratory disease were shown to be reflected by changes in tidal breathing patterns (Cain and Otis 1949). A change in respiratory rate (RR) or other breathing patterns can be indicative of a particular disease state and, as such, assessment of these features may result in a diagnosis (Braun 1990).

Historically, many features or parameters have been extracted from tidal breathing traces. In their simplest form, tidal breathing timing indices such as RR, inspiratory time (tI), expiratory time (tE), total breath time (tTot), and their ratios tI/tE and tI/tTot (duty cycle), provide an estimate of breathing frequency and asymmetry of tidal breaths. In addition, more complex parameters such as TEF50 (tidal expiratory flow at 50% of tidal volume), TIF50 (tidal inspiratory flow at 50% of tidal volume), and IE50 (inspiratory to expiratory flow at 50% tidal volume = TIF50/TEF50) can also be used to quantify the shape of tidal breathing flow–volume loops (Stocks 1996; Kaplan et al. 2000).

Respiratory rate is a vital sign used to monitor progression of illness and an abnormal RR can be an important marker of serious illness. There is substantial evidence that alterations in RR can be used to predict potentially serious clinical events such as cardiac arrest and may lead to unplanned admission to the intensive care unit (Fieselmann et al. 1993; Hodgetts et al. 2002; Cretikos et al. 2008). Other timing indices and their ratios have been helpful in elevating understanding of respiratory pathophysiology. For example, they have been used in the analysis of disease severity in chronic obstructive pulmonary disease (COPD) and cystic fibrosis (Colasanti et al. 2004), and more commonly in the assessment of infant respiratory conditions (Stocks 1996; Leonhardt et al. 2010). Colasanti et al. (2004) found that tI, tE, and tTot were all significantly shortened in patients with severe airway obstruction. In a more recent study comparing COPD patients with a cohort of healthy controls, they also reported a significantly shorter inspiratory, expiratory, and total breath time in the COPD group (Williams et al. 2014). Various other studies have shown the clinical utility of tidal timing indices/ratios (Morris and Lane 1981; Abramson et al. 1982; Carlsen and Lodrup Carlsen 2010) while others have provided evidence to suggest that parameters describing the flow–volume loop are also modified in different disease states (Papiris et al. 2002; Totapally et al. 2002; Tauber et al. 2003; de Boer et al. 2014). For instance, Carlsen and Lodrup Carlsen (1994) have shown that some tidal breathing parameters differ significantly between preschool children with and without asthma, and that some parameters also change in response to a bronchodilator.

Tidal breathing is commonly quantified by measuring oral/nasal airflow at airway openings. Pneumotachography (PNT) directly measures pressure at the mouth. From this pressure signal, flow may be estimated and, using numerical integration, a volume signal over time obtained. PNT is often used with a mouthpiece or a face mask and a tight seal around the mouth/mask is required for reliable PNT data acquisition. PNT is considered to be the gold standard against which other methods for respiratory measurement of tidal breathing are validated (Stick et al. 1992; Adams et al. 1993).

Tidal breathing over time can also be measured as movement of the thoraco–abdominal (TA) wall using devices such as respiratory inductive plethysmography (RIP) bands or piezoelectric belts that use transducers to convert TA movement into changes in voltage. Structured light plethysmography (SLP) is a recently developed technique that also measures TA wall movements during tidal breathing but one that utilizes a different technology to RIP. In SLP, movement of a projected grid of light on the anterior TA wall is recorded by two digital video cameras. Distortion of the grid through motion is then translated using previously reported algorithms to estimate anterior displacement of the TA regions and to generate a TA wall displacement‐over‐time trace (de Boer et al. 2010). The movement‐over‐time trace is derived from the average axial displacement of the TA wall. SLP is noninvasive, noncontact, and requires minimal patient/subject cooperation. It can be performed in infants and preschool children as well as the elderly and it can ultimately provide a dynamic high‐resolution (spatial) image of TA wall displacement over time. Although SLP does not measure flow or volume, TA displacement and TA displacement rate (i.e., the first derivative of TA displacement), respectively, can be considered similar conceptually. As a result, parameters broadly analogous to those obtained from PNT‐derived flow–volume loops can be calculated with this technique.

The use of SLP in clinical practice could potentially allow the use of tidal breathing respiratory parameters to be used more commonly for diagnosis, and initial indications from recent studies are promising. For example, Hmeidi et al. (2016a,b) demonstrated that IE50_SLP_ is significantly higher in children with acute and stable asthma when compared with healthy controls of a similar age. In addition, tI was found to be significantly shortened in COPD patients compared with an age‐, gender‐, and BMI‐matched cohort of healthy controls using SLP (Iles et al. 2015; Motamedi‐Fakhr et al. 2016). SLP has also been used to monitor tidal breathing parameters during the recovery of patients who have undergone a lung resection operation (Elshafie et al. 2016).

Structured light plethysmography has yet to be validated against the gold standard technique for measuring tidal breathing. The aim of this study was, therefore, to compare tidal breathing indices (RR, tI, tE, tTot, tI/tE, and tI/tTot) extracted from respiratory signals measured simultaneously using PNT and SLP in a diverse cohort of participants, as an initial step to validate SLP for more widespread clinical use. In addition, we examined IE50, as some evidence has suggested that this tidal breathing parameter may be a potential indicator of airway obstruction (de Boer et al. 2014; Iles et al. 2015; Hmeidi et al. 2016a,b; Motamedi‐Fakhr et al. 2016). To further test the robustness of the agreement between PNT‐ and SLP‐measured parameters, a separate cohort of healthy participants was recruited to examine the test–retest repeatability and the effect of a change in respiratory pattern and/or rate on the agreement between the devices. If SLP can perform equivalently to PNT in measuring clinically relevant aspects of tidal breathing, it may offer a less invasive methodology for obtaining such data.

## Methods

This was a nonrandomized pilot study. The study protocol was approved by the UK Health Research Authority National Research Ethics Service (study number 11/EE/00/37). All participants were fully informed of all testing protocols and provided written informed consent.

### Subjects

Twenty participants with a range of physician‐diagnosed respiratory conditions, as well as those with no previous or current respiratory diagnosis (the “primary cohort”) were recruited to evaluate the agreement between PNT and SLP in measuring tidal breathing parameters. Diversity among participants was desirable to provide a wide range of tidal breathing rates and respiratory patterns. No particular respiratory disorder was therefore excluded from the study. Twenty‐one additional healthy subjects with no previous diagnosis of a respiratory condition (the “repeatability cohort”) were recruited in order to assess the repeatability of the agreement between SLP and PNT. Participants from both cohorts were excluded if they had cold or any other viral infection, chest surgery within the past month, or an acute disease process likely to interfere with data acquisition.

### Study devices

Tidal breathing was recorded simultaneously using PNT (Viasys Masterscope CT; Viasys Healthcare GmbH, Höchberg, Germany) and SLP (Thora‐3Di^™^; PneumaCare Ltd, Cambridge, UK). The PNT device used had a sampling rate of 100 Hz, flow range of 0 to ±20 L/sec, and flow accuracy of ±2%. This device could generate a volume‐over‐time signal by digitally integrating the flow estimate; the volume range was specified to be 0 to ±20 L with a resolution of 1 mL. No calibration of PNT is required for measurement of tidal breathing timing indices and IE50; however, for this study, the PNT was calibrated in compliance with product instructions.

**Figure 1:**
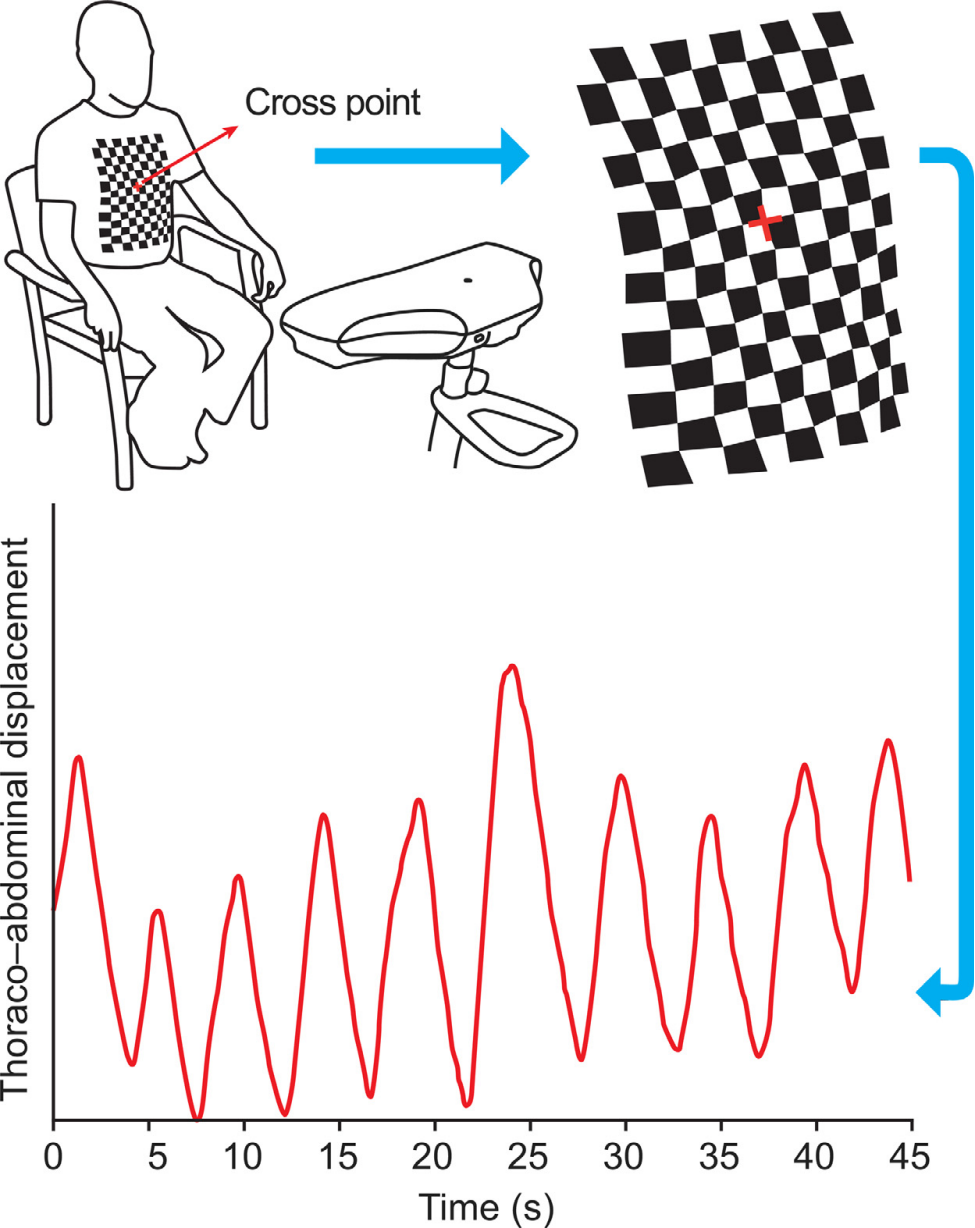
Structured light plethysmography projects a grid of light onto the thoraco–abdominal (TA) wall of a participant. The cross point of the grid is centered at the base of the xiphisternum. Changes in the grid pattern are recorded using two cameras (located in the scanning head) and then translated into a virtual surface corresponding to the shape of TA wall of the subject. Average axial displacement of the virtual grid provides a one‐dimensional movement–over‐time trace from which tidal breathing parameters can be calculated. Modified from Elshafie et al. 2016. s, seconds.

Figure 1 illustrates the working principle of SLP. The SLP device has a sampling rate of 30 Hz, which is sufficient to capture the dynamics of TA wall displacement. The grid pattern projected by the SLP device could be adjusted to accommodate the size of each participant's TA region. For this study, three different grid sizes (14 × 10, 12 × 8, and 10 × 6) were used.

### Study protocol

At the time of each measurement session, participants were asked to change into a close fitting white t‐shirt that followed the contours of the body or, if they preferred, SLP data acquisition could be performed on the bare chest. Participants were asked to sit upright in a high‐backed chair and to point to the bottom of their breast bone (xiphisternum). The projected grid was then aligned by the operator so that the center of the grid (the cross point) was positioned at the base of the xiphisternum, such that the grid covered an equal area above and below the xiphisternum from the clavicles to the anterior iliac crests.

To initialize the PNT measurement, participants were provided with a soft nose clip and were instructed to wear it in a manner that let no air out of the nasal passage. Participants were asked to tightly close their lips around a bacteriological filter‐mouthpiece connected to the PNT device. For each test scenario, after an initial familiarization period of approximately 15 sec, a 45‐sec epoch of tidal breathing data was acquired using both devices simultaneously. All comparisons were made using these 45‐sec epochs of data.

The primary cohort underwent one simultaneous resting measurement session on a single occasion to allow examination of the agreement between the two devices. The repeatability cohort underwent three measurement sessions, which were performed on the same day, by the same operator, in the same location, and using the same devices. After each measurement session, the participant was removed from the devices, requiring the device set‐up to be reinstated prior to the next measurement session. The first two measurement sessions were performed during resting tidal breathing 10–15 min apart (REST1 and REST2). Comparison of the 45 sec of data from REST1 and REST2 allowed examination of the repeatability of the agreement. The third measurement session was performed during recovery from an exercise‐induced elevation in RR. A Chester step test (CST) was carried out by the participant to achieve 80% maximum heart rate and an associated increase in RR (Sykes 1998). Immediately post exercise, the subject was seated and the third measurement session was performed (POST‐CST). Comparison of data from the combined REST1 and REST2 sessions with that from POST‐CST allowed assessment of whether a change in RR or respiratory pattern affected the agreement between the two devices.

### Software and data analysis

#### Preprocessing

Flow and volume time series from the PNT for each participant were exported using the Jscope software (Viasys Healthcare GmbH). Similarly, time series corresponding to the motion of the full anterior TA wall generated from SLP were exported from Pneumaview‐3D^™^ software (PneumaCare Ltd). Due to the difference in sampling frequencies for the two devices (PNT: 100 Hz; SLP: 30 Hz), the TA wall movement signal for each participant was resampled to match the sampling rate of the PNT signals using a piecewise cubic Hermite interpolating polynomial. This interpolation routine only minimally affects the signal morphology (i.e., it is shape‐preserving) and is therefore an appropriate choice of interpolant (Kahaner et al. 1989). Interpolating the SLP signal can change the position of its peaks/troughs by a maximum of 5 msec, which is considered negligible here.

**Figure 2:**
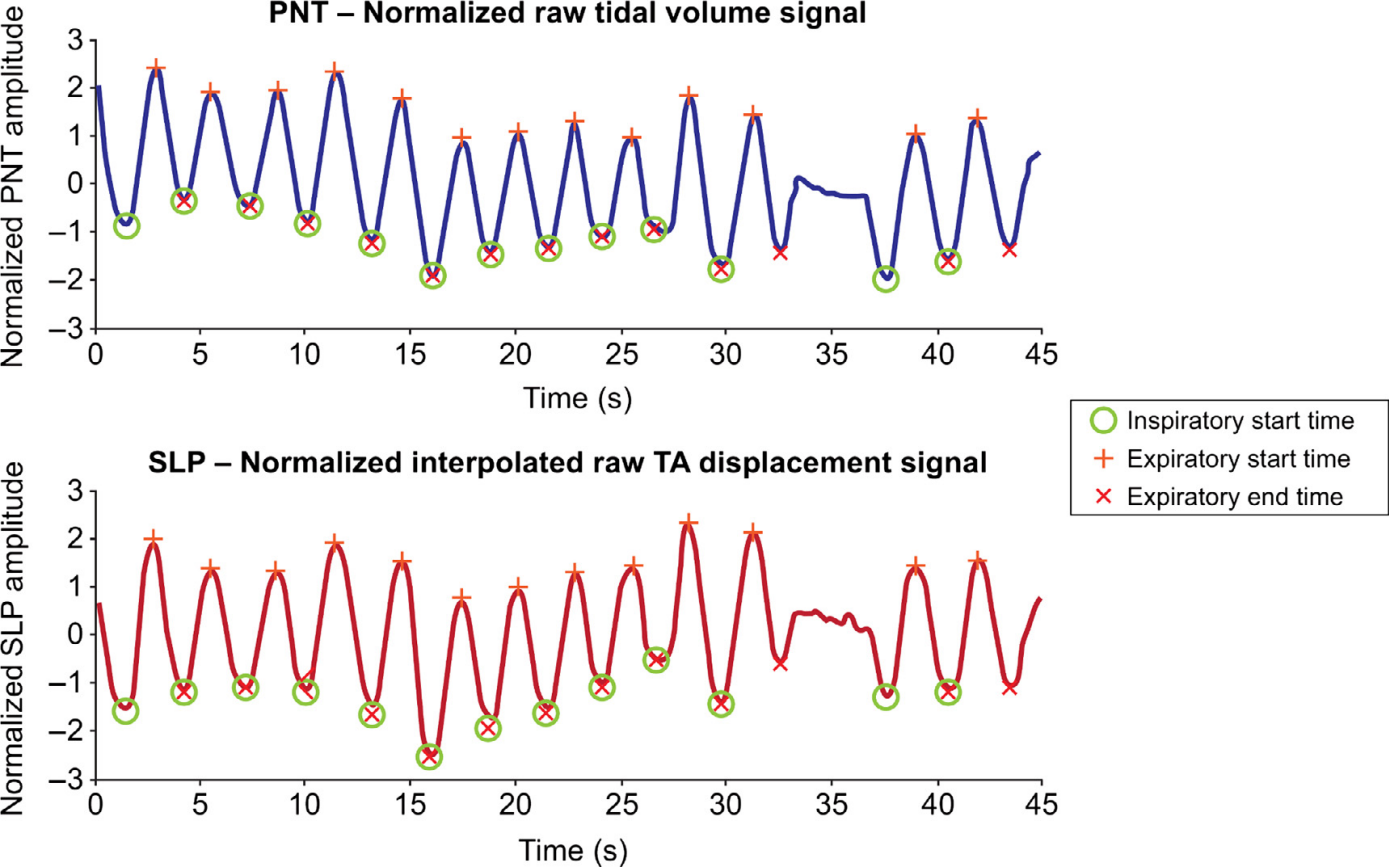
A comparison between PNT and SLP traces with marked breaths. Data displayed are from a healthy 8‐year‐old female. Although different in detail, traces are similar in morphology. Both traces are normalized (zero mean, unit standard deviation) for ease of visual comparison. Neither traces were filtered. Inspiratory start times (green circle), expiratory start times (red plus sign), and expiratory end times (red cross) are identified. Abnormal (artifactual) breaths are manually excluded (e.g., the breath at 35 s). Note that there is a one‐to‐one correspondence between the identified breaths, that is, the *n*th breath on the PNT corresponds to the *n*th breath on the SLP trace. PNT, pneumotachography; s, seconds; SLP, structured light plethysmography; TA, thoraco–abdominal.

Since the data acquisition procedures for PNT and SLP were synchronized manually by the operator, there were instances where the two signals were not perfectly aligned. For a breath‐by‐breath analysis, there was therefore a need to temporally align the shared portion of data. Since the morphology of the two traces was very similar, the peak of their cross‐correlation function was used for alignment. Figure 2 displays a comparison between a pair of PNT and SLP traces. Signals were band‐pass filtered prior to calculation of their cross‐correlation as baseline drifts can substantially affect the location of the cross‐correlation peak. However, this filtering was only done to align the two traces and not for calculation of the timing indices. Calculation of timing indices from unfiltered signals is preferable as filtering can introduce artifacts that affect the timing indices. The filter used for both traces was a fifth order band‐pass elliptic filter with pass‐band ripple of 0.5 dB and a stop‐band 50 dB down from the peak of the pass‐band. Pass‐band edges of the filter were set at 0.05 and 10 Hz. Traces were filtered in forward and reverse directions to avoid any relative phase shift. Cross‐correlation between each pair of filtered traces was calculated and location of the cross‐correlation peak was used to align the traces. All trace pairs were visually assessed to confirm that the process appeared reasonable. The peak of the cross‐correlation function was found to correspond to the point of best synchrony for all but one pair of traces, for which synchronization was achieved manually. Once aligned, traces were truncated to contain exactly the same number of samples.

Timing indices were calculated from the volume‐over‐time trace of PNT and the TA wall displacement‐over‐time trace of SLP. IE50 was calculated from the plot of flow versus volume and inspiratory to expiratory TA displacement ratio (IE50_SLP_) from a plot of TA displacement versus TA displacement rate.

#### Breath detection

A breath detection algorithm partially based on the previous works described by Bates et al. (2000)) and Schmidt et al. (1998) was used to assist with the breath identification process. The algorithm detected the peaks and troughs of the respiratory signal (i.e., TA movement‐over‐time or volume‐over‐time) using zero crossings of its first derivative. The identified peaks and troughs were visually assessed; those that did not correspond to inspiratory troughs or expiratory peaks were removed. A one‐to‐one correspondence between the identified breaths of the PNT and SLP signals was also established and visually verified. Figure 2 provides an example for clarification.

#### Calculation of tidal breathing parameters

**Figure 3:**
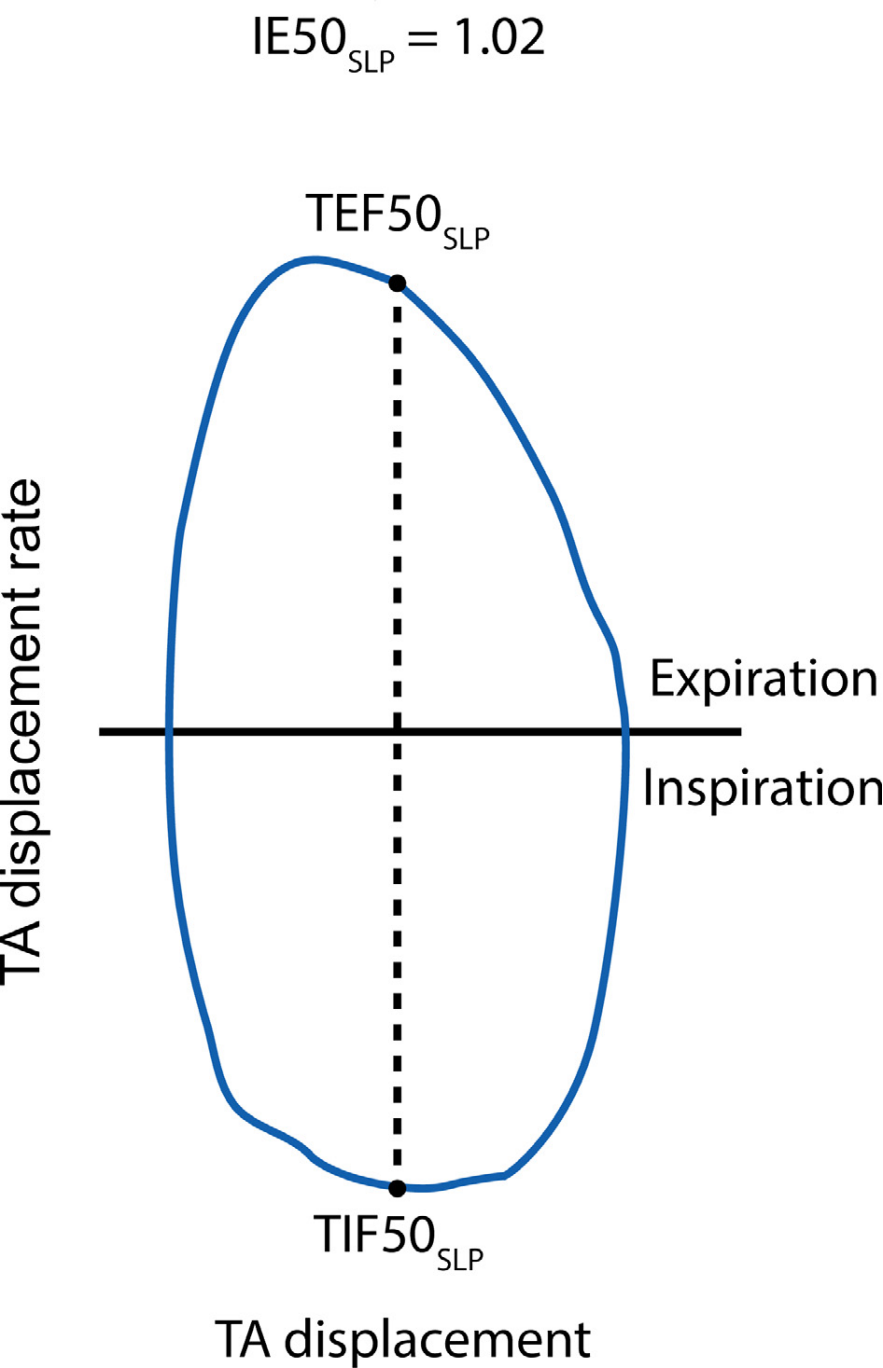
IE50_SLP_ is the ratio of TIF50_SLP_ to TEF50_SLP_ calculated from the displacement of the TA wall and its first derivative. Modified from Motamedi‐Fakhr et al. 2016. IE50, inspiratory to expiratory flow at 50% tidal volume; SLP, structured light plethysmography; TA, thoraco–abdominal; TEF50, tidal expiratory flow at 50% volume; TIF50, tidal inspiratory flow at 50% volume.

Inspiratory start times, expiratory start times, and expiratory end times, as shown in Figure 2, were identified. Inspiratory start time marks the start of an inspiration and is defined as a trough (a local minimum) on the volume–time or TA movement–time trace. Expiratory start time marks the start of an expiration, and is defined as a peak (local maximum) on the volume‐time or TA movement‐time traces. Expiratory end time marks the end of an expiration, and is defined as a trough that follows after expiratory start time on the respiratory trace. Defining a respiratory cycle in this manner is advantageous in calculating timing indices and also helpful in appraisal of individual breaths. Based on the above, and in compliance with the definitions by Stick (1996), for each breath, tI is obtained by subtracting inspiratory start time from expiratory start time, tE is found by subtracting expiratory start time from expiratory end time, and tTot is the difference between expiratory end time and the previous inspiratory start time. RR for each breath is defined as 60/tTot. The ratios tI/tE and tI/tTot are derived from the core parameters described above. IE50, defined as TIF50/TEF50, was calculated from the PNT flow signal. IE50_SLP_ was calculated as TIF50_SLP_/TEF50_SLP_ where TEF50_SLP_ is tidal expiratory TA displacement rate at 50% of expiratory displacement and TIF50_SLP_ is tidal inspiratory TA displacement rate at 50% of inspiratory displacement. IE50 and IE50_SLP_ are illustrated visually in Bates et al. (2000) and below in Figure 3, respectively.

### Statistical analysis

#### Primary cohort

Breath‐by‐breath differences between the calculated tidal breathing parameters were plotted in Bland–Altman charts (Bland and Altman 1986). Limits of agreement (LOA) for each parameter pair were calculated as “mean ± 1.96 × SD” of their difference. Further, Bland–Altman comparisons were made between the parameters averaged over 45 sec. Given that there were 20 participants in the primary cohort, Bland–Altman plots depicting the comparison between averaged parameters therefore contain 20 points. Note that since PNT is considered the gold standard for tidal breathing measurement, parameters measured by it are assumed to reflect the true values, and the *x*‐axes on the Bland–Altman plots therefore show the parameter value for the PNT as opposed to the average of PNT and SLP (Krouwer 2008). Pearson linear correlation coefficients between the two devices are also given for each parameter.

#### Repeatability cohort

To assess the repeatability of the agreement between the two devices, the 95% LOA for REST1 and REST2 were compared. Specifically, the equality of the upper and lower limits were simultaneously tested using a Wald test, assuming a significance level of 0.05 (e.g., see Harrell 2001a,b). To assess the effect of a change in RR or respiratory pattern on the agreement between devices, the combined agreement in REST1 and REST2 (Combined REST) was compared with that of POST‐CST. In a similar manner, equality of the lower and upper LOA were tested using a Wald test with a significance level of 0.05. Combining REST1 and REST2 provided a more accurate estimate of the mean and SD due to the larger sample size; however, having more observations did not bias the estimates.

For the averaged parameters, LOA were calculated using the “mean ± 1.96 × SD” of the difference between the two devices. For the breath‐by‐breath parameters, however, a repeated measures general linear model (with random subject effects) was used to estimate the overall mean bias and the LOA. To determine the magnitude of change in the LOA that would be detectable (with 80% probability), post hoc power analysis was performed on both breath‐by‐breath and averaged parameters.

## Results

### Agreement between SLP and PNT (primary cohort)

In the primary cohort, there were 20 subjects (13 male:7 female) with an age range of 6–78 years; mean (SD) 52 (23.5) years. A summary of demographics for the primary cohort is provided in Table 1. In total, 208 breath pairs were detected across all participants and were used for the breath‐by‐breath comparison. To compare averaged tidal breathing, each parameter was averaged over its time span of 45 sec. The average number of respiratory cycles for each subject was mean (SD) 10.4 (4.7). The minimum number of respiratory cycles across all subjects was five and the maximum was 20. Table 2 gives an indication of the distribution of the parameters in the primary cohort as measured using the gold standard (PNT).

**Table 1:** Demographics of the primary cohort

Respiratory status^a^	*N*	Individual ages	Gender
Healthy	4	[6, 8, 10, 54]	2M: 2F
COPD	6	[35, 43, 63, 69, 72, 78]	5M: 1F
Asthma	4	[49, 57, 72, 75]	2M: 2F
Pneumonia	2	[57, 64]	2M: 0F
Cystic fibrosis	1	8	0M: 1F
Sarcoidosis	1	65	0M: 1F
Suspected pulmonary edema	1	72	1M: 0F
History of pneumothorax	1	55	1M: 0F
Total	20	52 ± 23.5^b^	13M: 7F

COPD, chronic obstructive pulmonary disease; F, female; M, male; SD, standard deviation.

a Clinician diagnosis.

b Value is mean ± SD.

**Table 2:** Distribution of tidal breathing parameters in the primary cohort^a^

Tidal breathing parameters	Median [min, max]
RR (brpm)	13.5 [8.7, 29.2]
tI (sec)	1.85 [0.90, 2.59]
tE (sec)	2.58 [1.04, 4.44]
tTot (sec)	4.54 [2.11, 6.95]
tI/tE	0.79 [0.56, 1.23]
tI/tTot	0.44 [0.36, 0.54]
IE50	1.17 [0.83, 2.15]

brpm, breaths per minute; IE50, inspiratory to expiratory flow ratio at 50% tidal volume; PNT, pneumotachography; RR, respiratory rate; tE, expiratory time; tI, inspiratory time; tI/tE, inspiratory/expiratory time ratio; tI/tTot, duty cycle; tTot, total breath time.

a Note that the entries are calculated using the averaged timing index for each subject measured with PNT.

#### Respiratory rate (RR)

**Figure 4:**
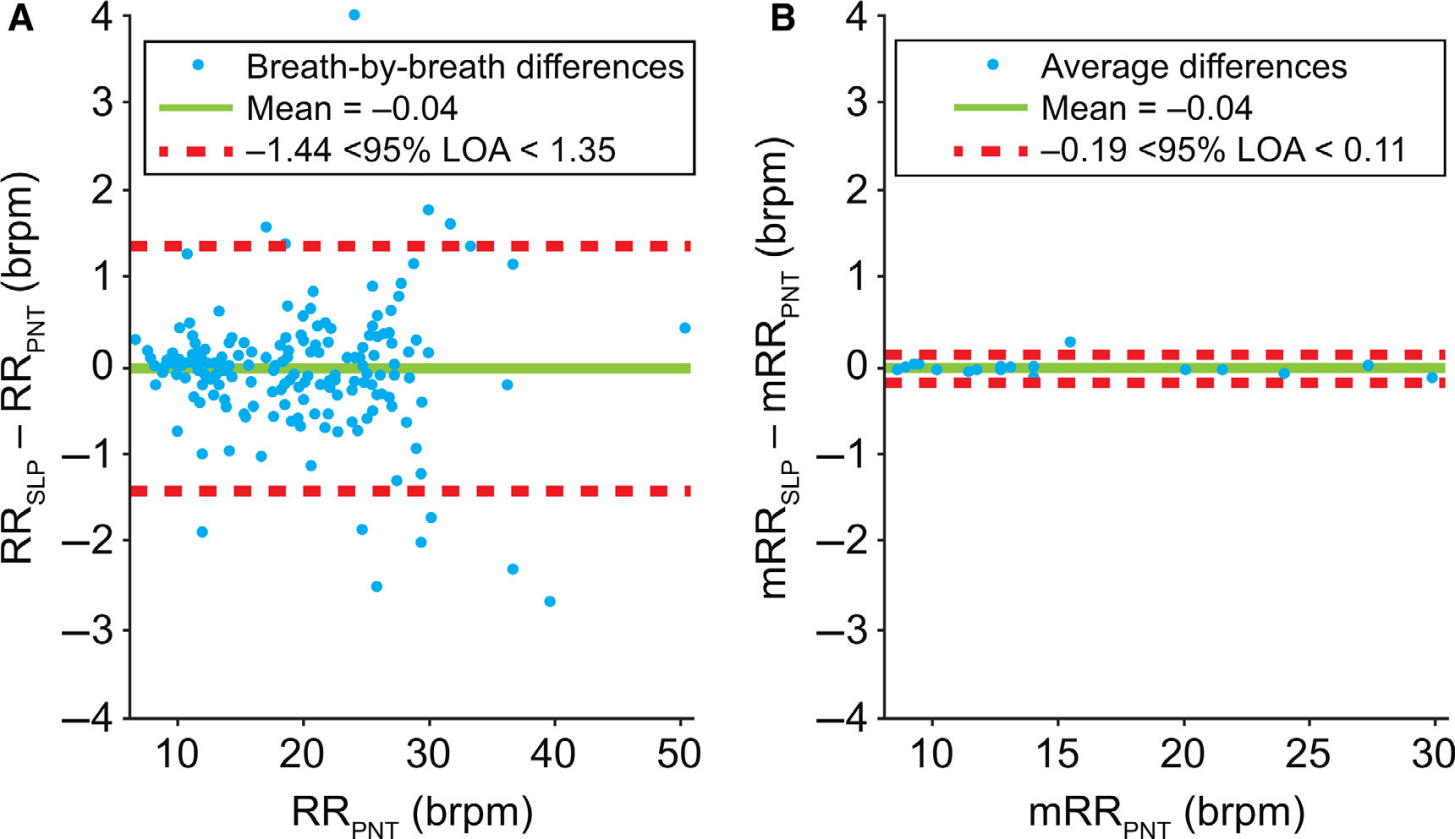
Comparison of (A) breath‐by‐breath and (B) averaged* respiratory rate between PNT and SLP. Note, a single point on the left graph is covered by the label. *Difference between the mean for each subject. brpm, breaths per minute; LOA, limits of agreement; m, mean; PNT, pneumotachography; RR, respiratory rate; SLP, structured light plethysmography.

Figure 4 depicts the agreement between PNT and SLP in measuring tidal breathing RR on both breath‐by‐breath and averaged levels. Unusually large/small RR values were retained to widen the range of agreement; however, 85% of the breath‐by‐breath RRs were within the range of 10–30 breaths per minute (brpm). The bias (mean) and 95% LOA are shown. Breath‐by‐breath agreement between PNT and SLP in measuring RR lay between −1.44 and 1.35 brpm. As expected, when averaged, the agreement improved with LOA spanning from −0.19 to 0.11 brpm. The mean difference in measuring RR was −0.04 brpm. The Pearson correlation coefficients for the breath‐by‐breath and averaged RR between PNT and SLP were 0.9953 and 0.9999, respectively. The scale on the *y*‐axes was intentionally kept identical between the two plots for ease of visual comparison. The distribution of differences in breath‐by‐breath and averaged RR were not found to be normal and a logarithmic transformation did not change that. Both distributions were more peaked than the normal distribution (kurtosis of 12.4 for the breath‐by‐breath distribution and 11.4 for the averaged) with the great majority of differences sitting around zero. The distributions were assessed using histograms and normal probability plots. Validity of the results despite the nonnormal distributions are considered further in the Discussion section.

#### Inspiratory time (tI)

**Figure 5:**
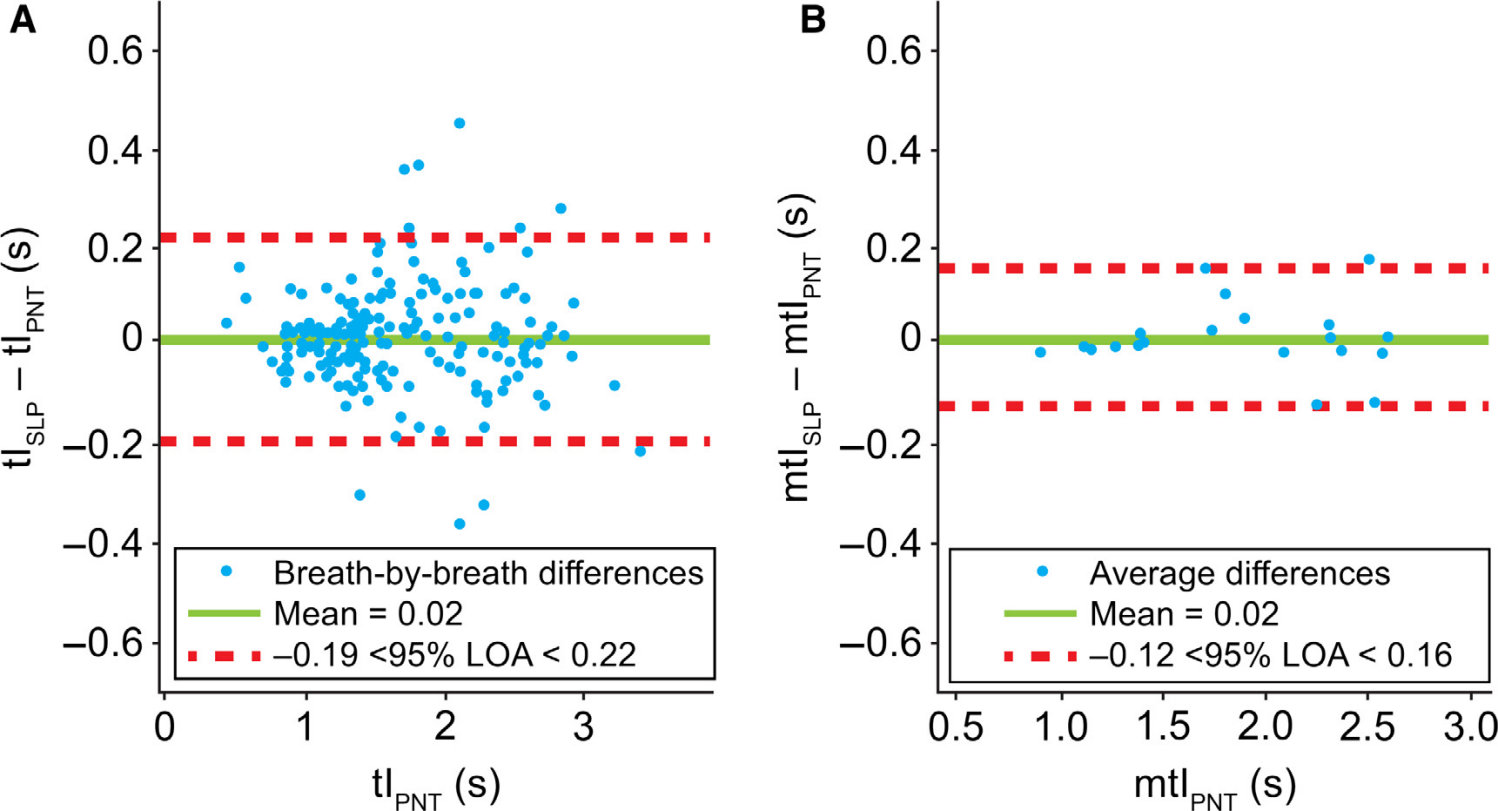
Comparison of (A) breath‐by‐breath and (B) averaged* inspiratory time between PNT and SLP. Note, a single point on the left graph is covered by the label. *Difference between the mean for each subject. LOA, limits of agreement; m, mean; PNT, pneumotachography; s, seconds; SLP, structured light plethysmography; tI, inspiratory time.

Figure 5 shows the agreement between PNT and SLP in measuring tI. For tI, LOA for the breath‐by‐breath comparison spanned from −0.19 to 0.22 sec. When averaged, LOA shrank to −0.12 to 0.16 sec. The mean bias was 0.02 sec for both comparisons. The Pearson correlation coefficients between tI_PNT_ and tI_SLP_ were 0.9844 and 0.9919 for the breath‐by‐breath and averaged measurements, respectively. Similar to RR, neither of the distributions were normal and a logarithmic transformation did not change that.

#### Expiratory time (tE)

**Figure 6:**
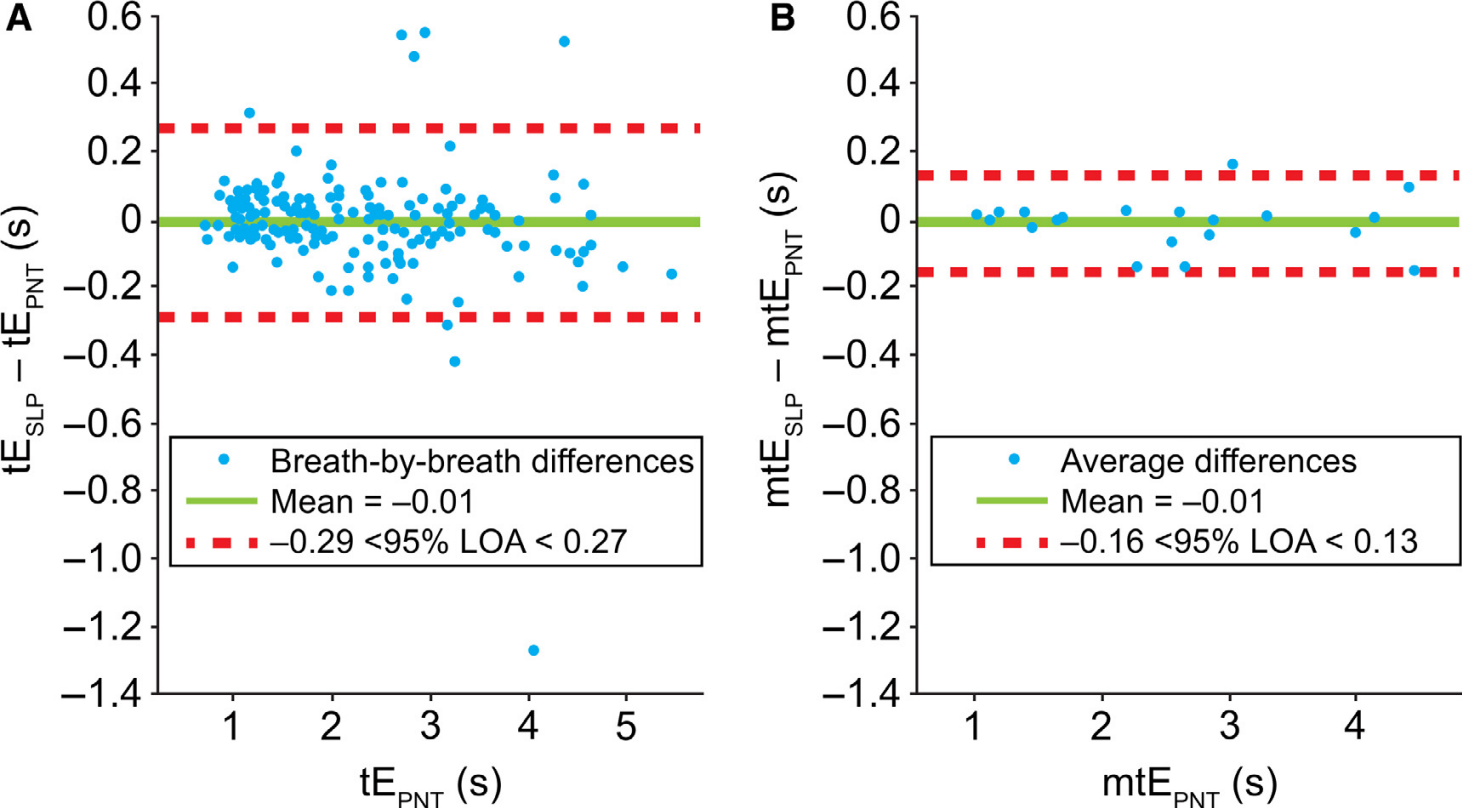
Comparison of (A) breath‐by‐breath and (B) averaged* expiratory time between PNT and SLP. *Difference between the mean for each subject. LOA, limits of agreement; m, mean; PNT, pneumotachography; s, seconds; SLP, structured light plethysmography; tE, expiratory time.

Figure 6 depicts the comparison between tE measured with the two devices. LOA for tE spanned from −0.29 to 0.27 sec; when averaged, LOA decreased to −0.16 to 0.13 sec. The bias sat around −0.01 in both cases. The Pearson correlation coefficients between tE_PNT_ and tE_SLP_ were 0.9902 and 0.9978 for the breath‐by‐breath and averaged measurements, respectively. The distributions of differences were not normal and, similar to the previous case, a logarithmic transformation did not change that.

#### Total breath time (tTot)

**Figure 7:**
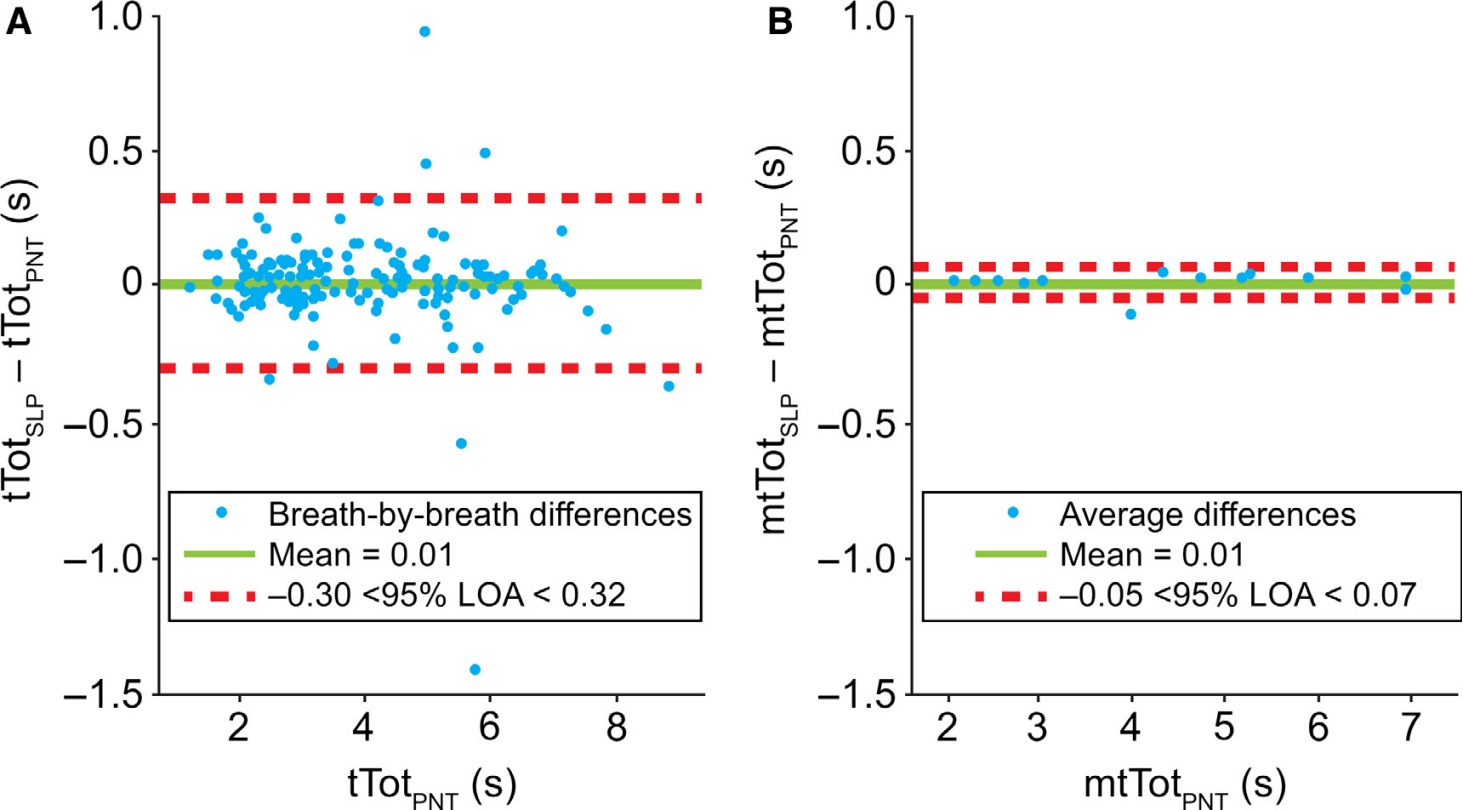
Comparison of (A) breath‐by‐breath and (B) averaged* total breath time between PNT and SLP. *Difference between the mean for each subject. LOA, limits of agreement; m, mean; PNT, pneumotachography; s, seconds; SLP, structured light plethysmography; tTot, total breath time.

Figure 7 depicts the comparison of tTot between the two devices. Breath‐by‐breath LOA extended from −0.30 to 0.32 sec. Once averaged, a considerably closer agreement was seen between the two devices, with LOA ranging from −0.05 to 0.07 sec. The bias remained similar (0.01 sec) for both graphs. The Pearson correlation coefficients between tTot_PNT_ and tTot_SLP_ were 0.9947 and 0.9998 for the breath‐by‐breath and averaged measurements, respectively. The distributions of differences were not normal and a logarithmic transformation did not lead to normality.

#### Inspiratory/expiratory time ratio (tI/tE)

**Figure 8:**
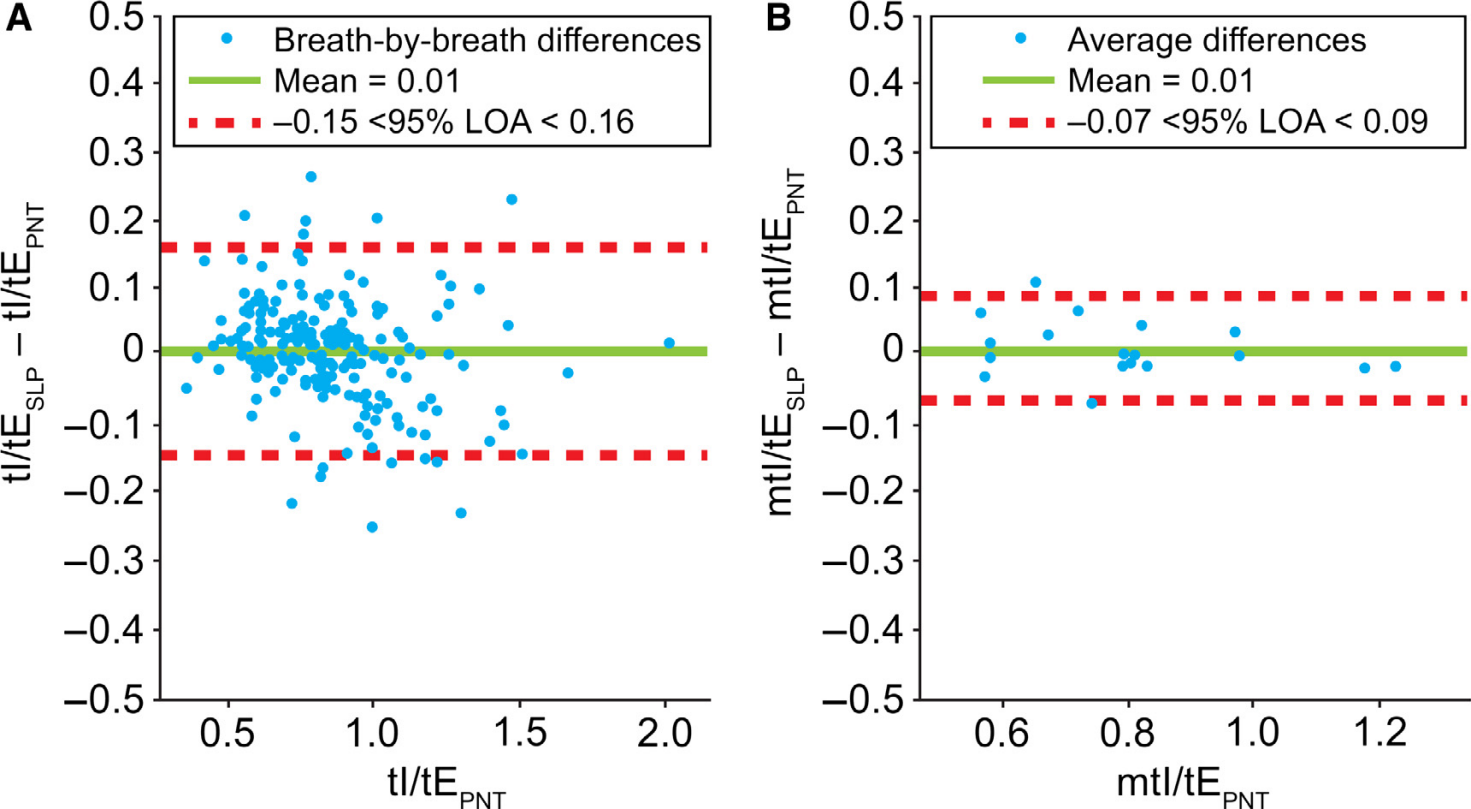
Comparison of (A) breath‐by‐breath and (B) averaged* inspiratory/expiratory time ratio (tI/tE) between PNT and SLP. *Difference between the mean for each subject. LOA, limits of agreement; m, mean; PNT, pneumotachography; SLP, structured light plethysmography; tE, expiratory time; tI, inspiratory time.

Figure 8 illustrates the agreement between tI/tE measured by the two devices. Inspiratory to expiratory ratios (I:E) between the two devices, as shown in Figure 8, had a LOA of −0.15 to 0.16 when compared on a breath‐by‐breath basis; when averaged, the LOA spanned from −0.07 to 0.09. The bias was 0.01. The Pearson correlation coefficients between tI/tE_PNT_ and tI/tE_SLP_ were 0.9481 and 0.9770 for the breath‐by‐breath and averaged measurements, respectively. The distribution of differences in breath‐by‐breath tI/tE was not normal and could not be logarithmically transformed to fit a normal distribution. The averaged differences in tI/tE, however, did conform to a normal distribution.

#### Duty cycle (tI/tTot)

**Figure 9:**
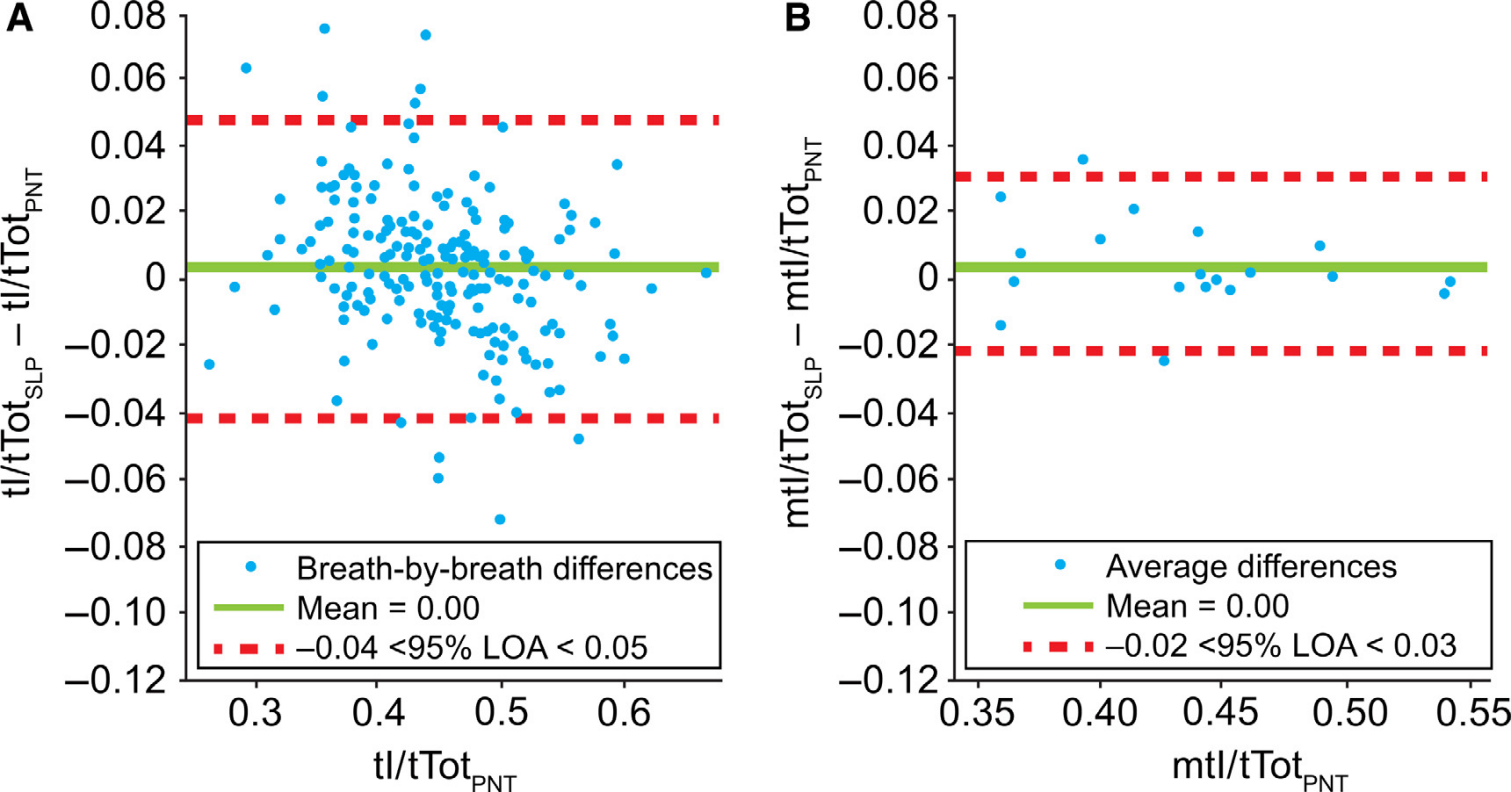
Comparison of (A) breath‐by‐breath and (B) averaged* duty cycle (tI/tTot) between PNT and SLP. Note, a single point on the left graph is covered by the label. *Difference between the mean for each subject. LOA, limits of agreement; m, mean; PNT, pneumotachography; SLP, structured light plethysmography; tI, inspiratory time; tTot, total breath time.

Figure 9 shows the agreement between the tI/tTot measured simultaneously using a PNT and SLP. LOA for tI/tTot had a lower agreement limit of −0.04 and an upper agreement limit of 0.05 (breath‐by‐breath); similar to previous cases, an improvement in agreement was observed by averaging the tI/tTot for each participant. LOA for averaged tI/tTot ranged from −0.02 to 0.03. The bias was found to be approximately zero. The Pearson correlation coefficients between tI/tTot_PNT_ and tI/tTot_SLP_ were 0.9418 and 0.9689 for the breath‐by‐breath and averaged measurements, respectively. The distribution of differences in breath‐by‐breath tI/tTot was not normal (and did not substantially change after a logarithmic transformation); the averaged differences, however, were found to approximate to a normal distribution.

#### IE50/IE50_SLP_

**Figure 10:**
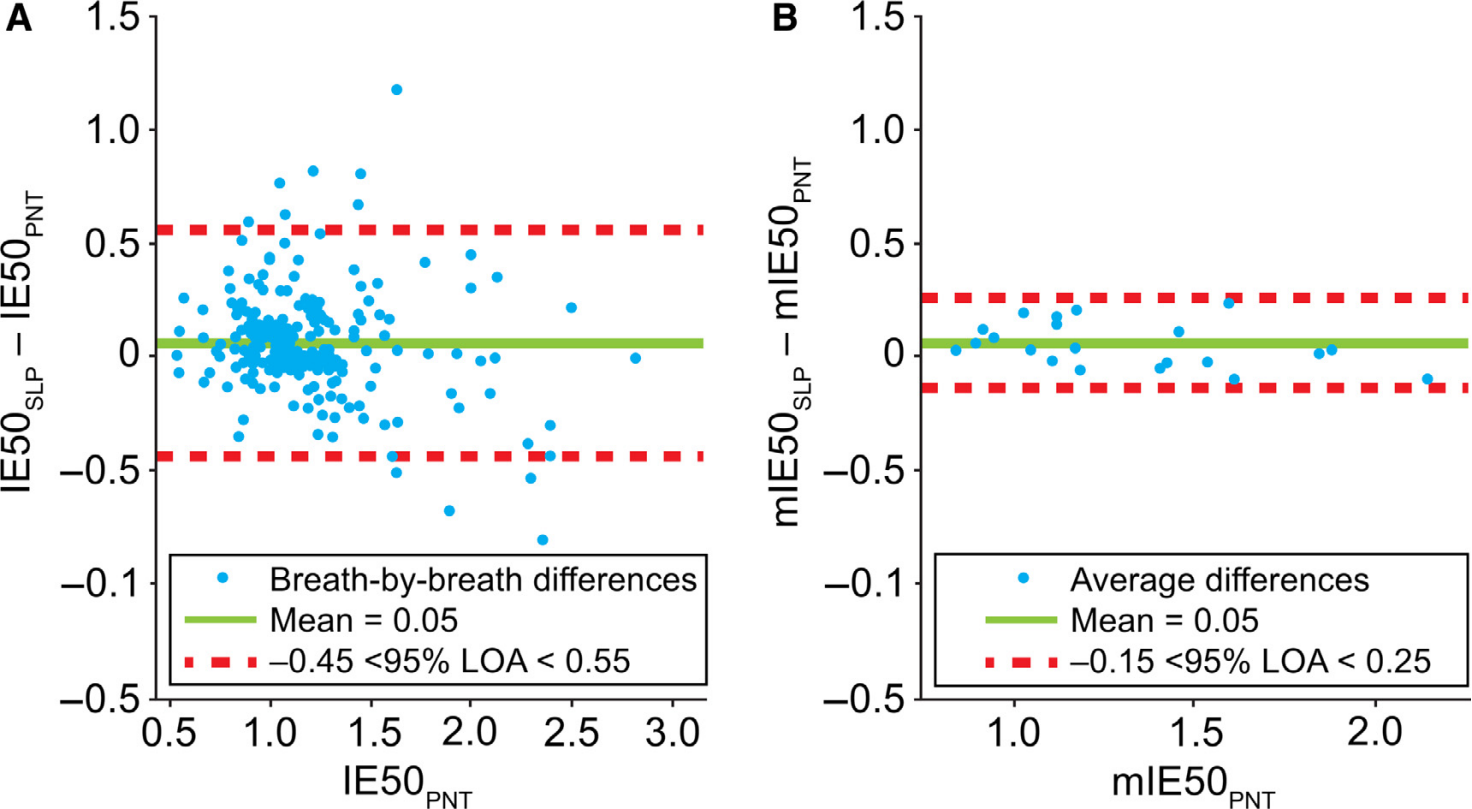
Comparison of (A) breath‐by‐breath and (B) averaged* IE50 and IE50_SLP_. Note, a single point on the left graph is covered by the label. *Difference between the mean for each subject. IE50, inspiratory to expiratory flow ratio at 50% tidal volume (or SLP equivalent; IE50_SLP_); LOA, limits of agreement; m, mean; PNT, pneumotachography; SLP, structured light plethysmography.

Figure 10 shows the agreement between IE50 and IE50_SLP_ measured simultaneously by PNT and SLP, respectively. LOA for IE50/IE50_SLP_ in the breath‐by‐breath comparison spanned from −0.45 to 0.55 with a bias of 0.05. Similar to previous cases, an improvement in agreement was observed by averaging IE50/IE50_SLP_ for each participant. LOA for averaged IE50/IE50_SLP_ ranged from −0.15 to 0.25. The bias remained the same (0.05). The Pearson linear correlation coefficients between IE50 and IE50_SLP_ were 0.8035 and 0.9623 for the breath‐by‐breath and averaged measurements, respectively. The distribution of differences in breath‐by‐breath IE50/IE50_SLP_ was not normal (and did not substantially change after a logarithmic transformation); the averaged differences, however, were found to approximately conform to a normal distribution.

#### All parameters

To provide an overview of the results, Tables 3 and 4 summarize the results as absolute and percentage differences between SLP and PNT. Bias and LOA for all parameter pairs are given. Percentage differences are defined as: (*X*_SLP_ − *X*_PNT_)/*X*_PNT_ where *X* can be any of the tidal breathing parameters included in the study.

**Table 3:** Absolute differences between tidal breathing parameters measured by PNT and SLP (primary cohort)

Tidal breathing parameters	Absolute difference between PNT and SLP (SLP–PNT)^a^	
	Breath‐by‐breath	Averaged (over 45 s)
RR (brpm)	−0.04 [−1.44, 1.35]	−0.04 [−0.19, 0.11]
tI (sec)	0.02 [−0.19, 0.22]	0.02 [−0.12, 0.16]
tE (sec)	−0.01 [−0.29, 0.27]	−0.01 [−0.16, 0.13]
tTot (sec)	0.01 [−0.30, 0.32]	0.01 [−0.05, 0.07]
tI/tE	0.01 [−0.15, 0.16]	0.01 [−0.07, 0.09]
tI/tTot	0 [−0.04, 0.05]	0 [−0.02, 0.03]
IE50	0.05 [−0.45, 0.55]	0.05 [−0.15, 0.25]

brpm, breaths per minute; IE50, inspiratory to expiratory flow ratio at 50% tidal volume (or SLP equivalent); LOA, limits of agreement; PNT, pneumotachography; RR, respiratory rate; SLP, structured light plethysmography; tE, expiratory time; tI, inspiratory time; tI/tE, inspiratory/expiratory time ratio; tI/tTot, duty cycle; tTot, total breath time.

a Values are mean [LOA].

**Table 4:** Percentage differences between tidal breathing parameters measured by PNT and SLP (primary cohort)

Tidal breathing parameters	Percentage difference between PNT and SLP (SLP–PNT)^a^	
	Breath‐by‐breath	Averaged (over 45 sec)
RR (%)	−0.13 [−7.7, 7.4]	−0.25 [−1.2, 0.7]
tI (%)	1.12 [−11.1, 13.4]	1.10 [−5.5, 7.7]
tE (%)	−0.10 [−11.0, 10.8]	−0.44 [−5.5, 4.6]
tTot (%)	0.27 [−6.8, 7.3]	0.15 [−1.2, 1.6]
tI/tE (%)	1.64 [−17.1, 20.4]	1.64 [−9.7, 13.0]
tI/tTot (%)	0.87 [−9.7, 11.4]	0.92 [−5.6, 7.4]
IE50 (%)	5.65 [−33.5, 44.8]	4.85 [−10.7, 20.4]

IE50, inspiratory to expiratory flow ratio at 50% tidal volume (or SLP equivalent); LOA, limits of agreement; PNT, pneumotachography; RR, respiratory rate; SLP, structured light plethysmography; tE, expiratory time; tI, inspiratory time; tI/tE, inspiratory/expiratory time ratio; tI/tTot, duty cycle; tTot, total breath time.

a Values are mean [LOA].

### Repeatability of the agreement between SLP and PNT (repeatability cohort)

In the repeatability cohort, there were 21 subjects (12 male:9 female) with an age range of 25–72 years; mean (SD) age 44.7 (14.7) years. Table 5 provides a summary of the distribution of parameters under each test condition in the repeatability cohort as measured by PNT.

**Table 5:** Distribution of tidal breathing parameters in the repeatability cohort^a^

Tidal breathing parameters	REST1	REST2	POST‐CST
	Median [min, max]	Median [min, max]	Median [min, max]
RR (brpm)	14.13 [10.02, 20.27]	14.56 [10.50, 19.90]	46.93 [26.90, 69.37]
tI (sec)	1.70 [1.22, 2.61]	1.76 [1.18, 2.54]	0.66 [0.45, 1.11]
tE (sec)	2.49 [1.63, 3.47]	2.27 [1.62, 3.60]	0.73 [0.42, 1.21]
tTot (sec)	4.27 [2.97, 6.01]	4.15 [3.05, 5.90]	1.35 [0.87, 2.24]
tI/tE	0.75 [0.52, 1.18]	0.82 [0.62, 1.04]	0.97 [0.76, 1.16]
tI/tTot	0.43 [0.34, 0.54]	0.45 [0.38, 0.51]	0.49 [0.43, 0.54]
IE50	1.20 [0.89, 2.07]	1.18 [0.86, 1.78]	0.99 [0.72, 1.39]

brpm, breaths per minute; IE50, inspiratory to expiratory flow ratio at 50% tidal volume; PNT, pneumotachography; POST‐CST, post exercise measurement session; REST1, resting breathing measurement session 1; REST2, resting breathing measurement session 2; RR, respiratory rate; tE, expiratory time; tI, inspiratory time; tI/tE, inspiratory/expiratory time ratio; tI/tTot, duty cycle; tTot, total breath time.

a Note that the entries are calculated using the averaged parameters for each subject measured with PNT.

Table 6 presents the mean bias and the 95% LOA for the REST1, REST2, Combined REST, and POST‐CST measurement sessions calculated from the breath‐by‐breath comparison. The *P*‐values testing the equality of the lower and upper LOA are also presented. For REST1 versus REST2, the *P*‐values were greater than 0.05 for all seven parameters, indicating that there was no significant difference in agreement between measurements during the two resting breathing sessions. Additionally, over a third of the rest period data (36%) had either between‐subject variances of zero or between‐subject variances that were less than 33% of the within‐subject variances. This attribute of the data meant that the LOA calculated using the repeated measures model were very close to the LOA calculated by pooling all data and were therefore comparable.

**Table 6:** Breath‐by‐breath agreement of tidal breathing parameters obtained using SLP and PNT during rest and post exercise measurement sessions (repeatability cohort)

Tidal breathing parameters	Breath‐by‐breath agreement		*P*‐value^a^ [lower, upper]
	Mean bias	LOA	
RR (brpm)			
REST1	0.019	−1.61, 1.65	[0.32, 0.24]
REST2	−0.015	−1.22, 1.19	
Combined REST	0.001	−1.42, 1.42	[0.03, 0.04]
POST‐CST	−0.045	−2.72, 2.64	
tI (sec)			
REST1	0.015	−0.28, 0.31	[0.65, 0.81]
REST2	0.023	−0.24, 0.29	
Combined REST	0.019	−0.26, 0.30	[<0.001, <0.001]
POST‐CST	0.011	−0.07, 0.09	
tE (sec)			
REST1	−0.016	−0.28, 0.25	[0.85, 0.80]
REST2	−0.014	−0.29, 0.26	
Combined REST	−0.015	−0.29, 0.26	[<0.001, <0.001]
POST‐CST	−0.008	−0.09, 0.07	
tTot (sec)			
REST1	−0.001	−0.37, 0.37	[0.99, 0.83]
REST2	0.009	−0.37, 0.39	
Combined REST	0.004	−0.37, 0.38	[<0.001, <0.001]
POST‐CST	0.002	−0.06, 0.06	
tI/tE			
REST1	0.016	−0.17, 0.20	[0.57, 0.61]
REST2	0.017	−0.14, 0.18	
Combined REST	0.016	−0.16, 0.20	[0.72, 0.55]
POST‐CST	0.022	−0.18, 0.22	
tI/tTot			
REST1	0.005	−0.05, 0.06	[0.60, 0.66]
REST2	0.006	−0.04, 0.05	
Combined REST	0.005	−0.05, 0.06	[0.94, 0.95]
POST‐CST	0.006	−0.05, 0.06	
IE50			
REST1	0.003	−0.66, 0.66	[0.75, 0.75]
REST2	0.060	−0.60, 0.72	
Combined REST	0.034	−0.63, 0.69	[0.21, 0.19]
POST‐CST	0.031	−0.43, 0.49	

brpm, breaths per minute; IE50, inspiratory to expiratory flow ratio at 50% tidal volume (or SLP equivalent); LOA, limits of agreement; PNT, pneumotachography; POST‐CST, post exercise measurement session; REST1, resting breathing measurement session 1; REST2, resting breathing measurement session 2; RR, respiratory rate; SLP, structured light plethysmography; tE, expiratory time; tI, inspiratory time; tI/tE, inspiratory/expiratory time ratio; tI/tTot, duty cycle; tTot, total breath time.

a *P*‐values test the equivalence of the lower and upper LOA in REST1 versus REST2 and Combined REST (REST1 and REST2) versus POST‐CST.

REST1 and REST2 were combined (Combined REST) and compared with POST‐CST using tests for equality of upper and lower LOA. For RR, the LOA were significantly wider for POST‐CST than for Combined REST (lower and upper *P*‐values = 0.03 and 0.04). For tI, tE, and tTot, the POST‐CST LOA were significantly narrower than for Combined REST (all *P*‐values < 0.001). The other parameters (tI/tE, tI/tTot, and IE50) did not show statistically significant differences in agreement between the rest periods and the post exercise period. Results of the post hoc power analysis for the breath‐by‐breath agreement are shown in Table 7. This shows the differences in LOA that were detectable with 80% probability (power) with this data and testing procedure. The minimum sensitivities ranged from 0.04 for tI/tTot to 1.1 sec for RR in the REST1 versus REST2 comparison, and from 0.04 for tI/tTot to 1.7 sec for RR for the Combined REST versus POST‐CST comparison.

**Table 7:** Difference in the upper or lower 95% LOA for breath‐by‐breath data that are detectable with 80% power^a^ (repeatability cohort)

Tidal breathing parameters	REST1 versus REST2	Combined REST versus POST‐CST
RR (brpm)	1.1	1.7
tI (sec)	0.2	0.2
tE (sec)	0.2	0.2
tTot (sec)	0.3	0.2
tI/tE	0.14	0.15
tI/tTot	0.04	0.04
IE50	0.5	0.45

brpm, breaths per minute; IE50, inspiratory to expiratory flow ratio at 50% tidal volume (or SLP equivalent); LOA, limits of agreement; POST‐CST, post exercise measurement session; REST1, resting breathing measurement session 1; REST2, resting breathing measurement session 2; RR, respiratory rate; tE, expiratory time; tI, inspiratory time; tI/tE, inspiratory/expiratory time ratio; tI/tTot, duty cycle; tTot, total breath time.

a Minimum detectable change using the current sample (80% power) and the breath‐by‐breath parameters.

Table 8 presents the mean bias and the 95% LOA for REST1, REST2, Combined REST, and POST‐CST measurement sessions calculated for the averaged parameters. The *P*‐values testing the equality of the lower and upper LOA are also presented. Upper and lower LOA were not significantly different for any of the parameters between REST1 and REST2. In Combined REST versus POST‐CST, both upper and lower LOA for tI, tE, and tTot were significantly narrower post exercise (*P* ≤ 0.01). There were no significant differences in the LOA of RR, tI/tE, tI/tTot, and IE50. Results of the post hoc power analysis for the agreement of averaged parameters are shown in Table 9. The minimum sensitivities ranged from 0.02 for tI/tTot to 0.26 for IE50 in the REST1 versus REST2 comparison, and from 0.02 for tI/tTot to 0.23 for IE50 in the Combined REST versus POST‐CST comparison.

**Table 8:** Agreement of averaged parameters obtained using SLP and PNT during rest and post exercise measurement sessions (repeatability cohort)

Tidal breathing parameters	Agreement of averaged parameters		*P*‐value^a^ [lower, upper]
	Mean bias	LOA	
RR (brpm)			
REST1	0.012	−0.23, 0.25	[0.85, 0.41]
REST2	−0.008	−0.22, 0.20	
Combined REST	0.002	−0.22, 0.22	[0.08, 0.42]
POST‐CST	−0.030	−0.34, 0.28	
tI (sec)			
REST1	0.017	−0.10, 0.13	[0.54, 0.96]
REST2	0.025	−0.08, 0.13	
Combined REST	0.021	−0.09, 0.13	[0.01, <0.001]
POST‐CST	0.011	−0.04, 0.06	
tE (sec)			
REST1	−0.017	−0.14, 0.10	[0.86, 0.91]
REST2	−0.016	−0.13, 0.10	
Combined REST	−0.016	−0.13, 0.10	[<0.001, 0.001]
POST‐CST	−0.008	−0.06, 0.04	
tTot (sec)			
REST1	0.000	−0.05, 0.05	[0.12, 0.85]
REST2	0.009	−0.03, 0.05	
Combined REST	0.004	−0.05, 0.05	[<0.001, <0.001]
POST‐CST	0.002	−0.00, 0.01	
tI/tE			
REST1	0.016	−0.08, 0.11	[0.80, 0.92]
REST2	0.018	−0.08, 0.11	
Combined REST	0.017	−0.08, 0.11	[0.46, 0.26]
POST‐CST	0.022	−0.10, 0.14	
tI/tTot			
REST1	0.005	−0.02, 0.03	[0.94, 0.81]
REST2	0.006	−0.02, 0.03	
Combined REST	0.005	−0.02, 0.03	[0.56, 0.46]
POST‐CST	0.006	−0.03, 0.04	
IE50			
REST1	0.002	−0.32, 0.32	[0.77, 0.36]
REST2	0.057	−0.29, 0.40	
Combined REST	0.029	−0.30, 0.36	[0.15, 0.19]
POST‐CST	0.034	−0.21, 0.28	

brpm, breaths per minute; IE50, inspiratory to expiratory flow ratio at 50% tidal volume (or SLP equivalent); LOA, limits of agreement; PNT, pneumotachography; POST‐CST, post exercise measurement session; REST1, resting breathing measurement session 1; REST2, resting breathing measurement session 2; RR, respiratory rate; SLP, structured light plethysmography; tE, expiratory time; tI, inspiratory time; tI/tE, inspiratory/expiratory time ratio; tI/tTot, duty cycle; tTot, total breath time.

a *P*‐values test the equivalence of the lower and upper LOA in REST1 versus REST2 and Combined REST (REST1 and REST2) versus POST‐CST.

**Table 9:** Difference in the upper or lower 95% LOA for averaged parameters that are detectable with 80% power^a^ (repeatability cohort)

Tidal breathing parameters	REST1 versus REST2	Combined REST versus POST‐CST
RR (brpm)	0.2	0.2
tI (sec)	0.1	0.1
tE (sec)	0.1	0.07
tTot (sec)	0.04	0.03
tI/tE	0.08	0.09
tI/tTot	0.02	0.02
IE50	0.26	0.23

brpm, breaths per minute; IE50, inspiratory to expiratory flow ratio at 50% tidal volume (or SLP equivalent); LOA, limits of agreement; POST‐CST, post exercise measurement session; REST1, resting breathing measurement session 1; REST2, resting breathing measurement session 2; RR, respiratory rate; tE, expiratory time; tI, inspiratory time; tI/tE, inspiratory/expiratory time ratio; tI/tTot, duty cycle; tTot, total breath time.

a Minimum detectable change using the current sample (80% power) and the averaged parameters.

## Discussion

Pneumotachography is a well‐established technique for measuring respiratory function that has been extensively used clinically and is considered by many as the gold standard for measurement of flow‐ and volume‐related indices extracted from tidal breathing (Stick 1996). SLP is a novel, noncontact, respiratory monitoring device, which measures the displacement of the TA wall. It is essential to note that PNT and SLP measure different features of respiration and, consequently, some discrepancy between the two devices is to be expected. A simultaneous comparison between the two devices was therefore carried out to establish how SLP compares with PNT in measuring tidal parameters.

Differences in RR of less than or equal to ±2 brpm are considered clinically insignificant (Smith et al. 2011). We adopted this threshold as our criterion for defining agreement between PNT and SLP. Differences between averaged RR measured with SLP and PNT were found to be more than an order of magnitude smaller than the tolerance of ±2 brpm over the entire range considered. Differences in breath‐by‐breath RRs were also within the tolerance. On this basis, RR measured using SLP was judged to be in agreement with RR measured with PNT for both the breath‐by‐breath analysis and the averaged RR comparison. For the other parameters, there appear to be no established clinical tolerance limits; however, some information can be extrapolated from the relationship between RR and tTot and their respective LOAs. The LOA for breath‐by‐breath tTot (−0.30 to 0.32) had the largest range for LOA among all the studied nonratio timing indices. Given that RR was found to be in agreement between the two devices over the range of measurement and that RR is directly derived from tTot, one might expect tTot to also be in agreement over the entire range. A tolerance of ±2 brpm between the two devices in RR predicts a tolerance in the magnitude of the difference in tTot of less than 0.3 sec as long as tTot is greater than 3 sec. When examining averaged tTot (LOA of −0.05 to 0.07 sec), a tolerance of ±2 brpm between the two devices in RR predicts a tolerance in the magnitude of the difference in tTot of less than 0.05 sec as long as tTot is greater than 1.2 sec. Percentage difference between PNT and SLP in measuring tTot was also very similar to that of RR. On that basis, it seems reasonable to judge that there was an agreement between PNT and SLP when measuring tTot over the whole measurement range for the averaged measurements, and for tTot above 3 sec for the breath‐by‐breath analysis.

From a different and probably more appropriate perspective, it is possible to consider tTot in its own context, and to answer the key question of whether a difference of ±0.3 sec between PNT and SLP in measuring tTot is clinically significant. As discussed, there is no established answer to this; however, it is not uncommon to see respiratory signals low‐pass filtered at 0.5 Hz prior to analyses (Chervin et al. 2008; Walter and Vaughn 2013). Such filtering can shift the inspiratory and expiratory markers by up to half a second, hence affecting tidal breathing indices. Note that this shift on the local extrema is not caused by lack of zero‐phase filtering, it is caused by excessive smoothing of the respiratory trace, which reduces physiological double or triple peaks to a single peak, changing peak location in the process. Although preprocessing a respiratory signal in such a manner can remove some of its embedded physiology (Motamedi‐Fakhr et al. 2013), it could also mean that a shift of approximately 0.5 sec is not considered to be clinically significant. If so, then not only tTot, but tI and tE can be deemed equivalent between the two devices (as all LOAs were well within ±0.5 sec) on a breath‐by‐breath and averaged basis over the entire measurement range. tI and tE both displayed narrower LOAs than tTot and this provides some further justification for the judgment that tI and tE measured with PNT and SLP were in agreement.

tI/tE has most often been reported in relation to mechanical ventilation. Again, we have been unable to find established normative values for tI/tE and its relative errors and, as a result, it is difficult to solidly justify the agreement here. When averaged, the LOA for tI/tE between PNT and SLP was within ±0.1. Studies involving mechanical ventilation commonly involve setting variable inspiration to expiration (I:E) ratios to improve patient ventilation. tI/tE is usually derived from dividing two integers; for example, 1:1, 1:2, and 1:4 are common I:E ratio settings for ventilators. Given the paradigm described, it seems unlikely that a discrepancy of 0.1 in I:E ratio is clinically significant. On a breath‐by‐breath level, however, discrepancies as large as 20% in tI/tE may be significant, although this would depend strongly on the specific application.

Duty cycle (tI/tTot) gave the narrowest LOA; this was expected as it also had the narrowest range of values. When averaged, the LOA ranged from −0.02 to 0.03; although seemingly very small, it is not clear whether this is clinically significant. In a similar study validating RIP against PNT, it was reported that tI/tTot, averaged over 10 breaths, had LOA spanning from −0.016 to 0.064 (Stick et al. 1992). It is worth noting that this previous study was performed in sleeping infants (quiet sleep) and only highly selected breaths were used in the comparison. The LOA for our comparison between SLP and PNT was slightly narrower and was roughly centered around zero (no bias), suggesting a superior agreement.

IE50 and IE50_SLP_ – two related parameters that describe the shape of the flow–volume and TA displacement–TA displacement rate loops, respectively, did show less agreement than the basic timing indices on a breath‐by‐breath level. However, when averaged, agreement improved considerably. Whether the observed agreement is clinically acceptable is not fully clear, although considering the high within‐subject variability of IE50/IE50_SLP_ (with the average SD of IE50 across all subjects being 0.27), a 95% LOA spanning from −0.15 to 0.25 does seem sufficiently narrow.

In terms of percentage differences, assuming that a percentage difference of less than 10% is not clinically significant, PNT and SLP were shown to agree in measurement of RR, tI, tE, tTot, and tI/tTot when averaged. Averaged tI/tE differed between the devices by slightly more than 10%, and IE50 differed by up to 20%. It is important to note, however, that an SLP data capture in standard use would typically last for 5 min rather than the 45 sec used in this study. It is likely that agreement will be further improved when averaged over this longer data collection period. This will be the subject of further investigation.

As the distributions of differences in the parameters were mostly not normal, and a logarithmic transformation did not change that, one might argue that LOA calculated using the mean and the SD is not representative of the true LOA. This is in fact true; however, according to Bland and Altman, LOA calculated this way tend to be further apart rather than too close and should not result in a false agreement (Bland and Altman 1999). It is therefore safe to state that the LOAs reported in this study are, in fact, conservative.

Even though there were inherent differences between the primary and repeatability cohorts, the bias and the LOA remained largely similar during rest; an indication of the reproducibility of the agreements. In the repeatability cohort, there was no significant difference in the agreement between devices from REST1 to REST2 in any of the seven parameters, again indicating good repeatability in agreement. During the post exercise (POST‐CST) test, RR was considerably raised compared with the rest periods (Combined REST). When Combined REST was compared with POST‐CST, the LOA for tI, tE, and tTot became narrower (tighter) on both the averaged and breath‐by‐breath levels. This was expected as breathing at a higher RR leaves less room for variation in breath timing components. Of all the comparisons made, the only significant reduction in agreement was observed for breath‐by‐breath RR, where the LOA widened. We suggest that the use of a constant ±2 brpm acceptance criterion for all RRs may be unduly restrictive. RR is a parameter derived from tTot (RR = 60/tTot), and rather sensitive to small changes in tTot when tTot itself is relatively short. The increase in LOA at higher RRs is therefore not necessarily an indication of poor agreement in this study, but may be an indication of an inappropriate choice of acceptance criterion. Hence, we take the view that, despite this diminished agreement at higher RRs, the agreement between devices can be considered to have remained adequately repeatable, since tTot showed a close agreement.

The results of the post hoc power analysis illustrated the minimum change in a parameter that can be detected with 80% likelihood using the current samples and set‐up. Of note were the averaged minimum sensitivities, which are lower than or equal to 0.1 sec for tI, tE, and tTot, 0.2 brpm for RR, and 0.26 for IE50. This was performed on 45 sec of data in 21 subjects. By increasing the length of measurement and/or the number of subjects, this sensitivity is likely to be improved.

Structured light plethysmography is sensitive to excessive movement and therefore cannot be used for conventional sleep studies (as subjects may move) or during exercise testing. However, the findings of this study suggest SLP is a useful new technique for measuring tidal breathing and can be used post exercise. Furthermore, unlike other methods, SLP does not require direct contact with the subject or the use of face masks, nose clips, bands, or belts, and, other than requiring them to sit still, requires minimal cooperation from the subject.

## Conclusion

In this study, breath‐by‐breath and averaged tidal breathing parameters (RR, tI, tE, tTot, tI/tE, tI/tTot, IE50) measured by PNT and SLP were compared. RR was found to agree very well between the two devices. There are strong indications that tI, tE, tTot, tI/tTot, and IE50 may also be judged to agree between PNT and SLP when averaged and, generally, also on a breath‐by‐breath level. Together with the evidence that SLP as a method is repeatable, the agreement in these fundamental parameters indicates that, despite the difference in technologies, SLP performs well as a measure of tidal breathing parameters when compared to the performance of the gold standard PNT.

## Conflict of Interest

RI was a part‐time paid medical advisor to PneumaCare Ltd at the time of the study and is currently a shareholder of PneumaCare Ltd. RCW and SMF are employees of and have share options for PneumaCare Ltd. WdB and AK were employees of PneumaCare Ltd at the time of the study. WdB holds shares in PneumaCare Ltd. AB and JC have declared no conflicts of interest, financial or otherwise.
